# A dose–response correlation between smoking and severity of acute pancreatitis: a propensity score-matched study

**DOI:** 10.3389/fmed.2024.1397111

**Published:** 2024-07-29

**Authors:** Runzhuo Li, Wanyun Tang, Sun Yan, Xiaohan Yu, Lian Hu

**Affiliations:** ^1^Department of Digestion, First People's Hospital of Yibin, Yibin, China; ^2^Department of Digestion, Dandong Central Hospital, China Medical University, Dandong, China; ^3^Department of Orthopedics, Dandong Central Hospital, China Medical University, Dandong, China; ^4^Department of Cardiology, The First Affiliated Hospital of Chongqing Medical University, Chongqing Medical University, Chongqing, China; ^5^General Surgery Department, Dandong Central Hospital, China Medical University, Dandong, China

**Keywords:** smoking, acute pancreatitis, propensity score-matching, severity grading, dose–response relationship

## Abstract

**Background:**

Acute pancreatitis, among the most prevalent gastrointestinal disorders, exhibits a continual rise in its incidence recent years. This study endeavor to explore the correlation between smoking exposure and the severity of acute pancreatitis (AP).

**Methods:**

Five hundred and eight patients diagnosed as acute pancreatitis (AP) were included in our data analysis. Patients were categorized based on their smoking pack-years into four groups: light, moderate, heavy, and non-smokers. Outcomes were classified as two: “mild acute pancreatitis (MAP)” and “moderately severe acute pancreatitis (MSAP) or severe acute pancreatitis (SAP)”. We conducted propensity score matching (PSM) to adjust confounding factors and multivariable logistic regression analysis to determine adjusted odds ratios and 95% confidence intervals. Additionally, a dose-dependent association analysis between smoking exposure and the incidence rate of “MSAP or SAP” was performed.

**Results:**

Smokers exhibited a higher risk of “MSAP or SAP” compared to non-smokers, both before (17.1 vs. 54.9%, *p* < 0.001) and after (9.4 vs. 24.7%, *p* < 0.001) PSM. With an area under the ROC curve of 0.708, smoking showed a moderate level of predictive ability. Furthermore, propensity score matching analysis showed that patients who smoked compared to non-smokers had significantly higher risks of “MSAP or SAP” for light smoking (OR 3.76, 95% CI 1.40–10.07, *p* = 0.008), moderate smoking (OR 4.94, 95% CI 2.23–10.92, *p* < 0.001), and heavy smoking (OR 8.08, 95% CI 3.39–19.25, *p* < 0.001).

**Conclusion:**

Smoking is an independent risk factor that can raise the severity of pancreatitis. Moreover, the severity of acute pancreatitis escalates in tandem with the accumulation of pack-years of smoking.

## Introduction

Acute pancreatitis (AP) is an intense inflammatory leading to edema, hemorrhage, and potentially necrosis (1, 2). With an incidence rate estimated at 110–140 cases per 100,000 population, AP has become one of the most prevalent gastrointestinal illnesses requiring hospitalization in the United States (3). Between 2002 and 2013, the number of hospitalizations attributed to AP rose from 9.48 per 1000 cases to 12.19 per 1000 cases (4). According to the 2012 Atlanta guidelines, AP fall under the category as mild (MAP), moderately severe (MSAP), or severe (SAP) in accordance with its severity of onset. The latter two categories, MSAP and SAP, are more severe and often lead to local or systemic complications including pancreatic leakage, pancreatic necrosis, pancreatic abscess, systemic inflammatory response syndrome (SIRS), even multi-organ dysfunction syndrome (MODS) (5). These complications not only prolong hospitalization and increase medical costs but may also result in long-term disabilities and mortality. Approximately 80% of patients present with MAP to MSAP, with one-fifth progressing to severe disease, and a mortality rate of about 20% (6–8). Traditional causes of AP include gallstones, alcohol abuse, and hypertriglyceridemia. Many studies (9–12) has reported smoking also accounting for the incidence of AP. However, within Asian populations, there is limited research on the relationship between smoking and AP, particularly regarding its impact on the severity of the disease.

Currently, smoking is a well-known risk factor for various digestive diseases (13). Nicotine and other harmful substances in tobacco smoke can cause pancreatic damage, inflammation, and impaired pancreatic function. Smoking has been shown to inhibit pancreatic secretion and increase the risk of leakage of pancreatic enzymes into the bloodstream. Prolonged exposure to nicotine increases the content of pancreatic zymogen within cells and induces vacuolization of acinar cells in rats (14). These factors may exacerbate the development of AP and its complications. Therefore, elucidating the link between smoking exposure and the severity of AP is crucial for preventing AP from progressing into an irreversible and lethal disease. The goal in our study is to figure out the link between smoking exposure measured in pack-years and the severity of AP through propensity score matching analysis.

## Methods

### Data sources

This retrospective investigation utilized anonymized clinical data from electronic medical records of patients admitted for AP at Dandong Central Hospital, China Medical University from October 2017 to October 2023. The Dandong Hospital Ethics Committee approved the study and granted a consent waiver for this retrospective cohort study. In order to protect personal health information, online medical records were limited to the range of anonymized data analysis, which included demographics, message of laboratory findings, imaging analysis results, and various complications.

### Data collection

In this study, we collected variables including demographic characteristics: age, gender, smoking history, drinking history, hypertension, diabetes, hyperlipidemia, cardiovascular disease; and serological indicators: white blood cell count (WBC), red blood cell count (RBC), hemoglobin concentration (HGB), platelet count (PLT), potassium ion (K+), sodium ion (Na+), calcium ion (Ca+), creatinine (Cr), blood glucose (BG), alanine aminotransferase (ALT), lactate dehydrogenase (LDH), total bilirubin (TBIL), albumin (ALB), amylase (AMY), lipopolysaccharide (LPS), D-dimer, C-reactive protein (CRP), prothrombin time (PT), and activated partial thromboplastin time (APTT).

All serological indicators for the patients were obtained from blood samples collected within 24 h of admission.

### Patient selection

Both the diagnosis and severity grading of AP referred to the 2012 Atlanta guidelines (5). We included patients in our study who satisfied at least two of the following criteria. (1) Acute, persistent, intense pain in the upper abdomen, with or without radiation to the back; (2) The activity of serum lipase (or amylase) beyond three times the upper limit of normal; (3) Imaging findings match characteristics of AP.

Patients were excluded from the study if meeting any of following criteria. (1) Presence of other pancreatic diseases, such as chronic pancreatitis; (2) Presence of other serious diseases, such as tumors, autoimmune diseases, etc.; (3) Incomplete or unavailable data; (4) Taking medications that may affect the severity of pancreatitis; (5) History of pancreatic surgery; (6) Previous hospitalization for AP.

Before the commencement of the study, four researchers received professional training in data collection, including the diagnosis and severity grading of AP. Three researchers independently collected the variables. If necessary, any differences in variable identification were discussed with or determined by the senior researcher.

#### Exposure and outcome

Our study's main exposure factor was smoking pack-years, calculated by multiplying the daily pack count by the total number of smoking years. The patients were categorized into smoking and non-smoking groups according to their smoking history. Smokers included in the study were those who had been continuously smoking and were still smoking within 1 month of disease onset. To assess the link between dosage and response of smoking pack-years and AP severity, study population was further split into four groups on the basis of pack-years: nonsmokers (Never smoking), smokers with pack-years ≤ 10 (Light smoking), smokers with pack-years >10 but ≤ 20 (Moderate smoking), and smokers with pack-years >20 (Heavy smoking).

The primary outcome assessed was the severity of AP, graded referring to the 2012 Atlanta guidelines. AP severity categorization was determined on patients' clinical complaints and symptoms, serological indicators, imaging findings, presence of organ failure, and complications, dividing AP into MAP, MSAP, and SAP. Based on previous research (15), we defined the outcome variable as “MAP” and “MSAP or SAP”, categorized as binary variables “yes” or “no”, for our statistical analysis.

#### Analytical statistics

When analyzing patients' baseline characteristics, various types of data necessitate specific methods. Percentages (%) are applicable to categorical variables, with the chi-square test used for group comparisons. Mean ± standard deviation (SD) is suitable for normally distributed continuous measurements, with independent sample *t*-tests employed for group comparisons. Median and interquartile range are utilized for non-normally distributed data, with the Mann-Whitney *U*-test used for group comparisons.

We investigate the linkage between smoking and the severity of AP using logistic regression modeling. After possible confounding factors with *p*-value ≥ 0.05 are excluded using univariable logistic regression, factors with *p*-value <0.05 are then included in the multivariable regression analysis. Additionally, we computed the area under the ROC curve (AUC) to ascertain discriminative capacity of smoking pack-years for distinguishing between “MAP” and “MSA or SAP”. Then we assessed the relationship between smoking pack-years and AP severity through graphical representations illustrating the association between observed rates and predicted probabilities across various levels of smoking pack-years.

To further mitigate the influence of confounding variables, a propensity score matching (PSM), of which matching ratio was 1:1, was performed by the nearest neighbor algorithm, to make sure the most appropriate balanced distribution of covariates between groups. With a caliper width of 0.25 standard deviations, the matching effect was assessed by standardized mean differences (SMDs). Following PSM, we stratified all covariates and then conducted univariable logistic regression analysis to explore the linkage between smoking pack-years and the severity of AP. Finally, for assessing the strength of the link. we calculated odds ratios (ORs) and 95% confidence intervals (CIs).

SPSS version 27 (IBM Corp., Armonk, NY, USA) and R software version 4.0.3 (R Foundation for Statistical Computing, Vienna, Austria) were our main statistical tools that we used.

## Results

### Baseline characteristics

Four hundred and twenty-one patients were disqualified in total based on the exclusion criteria. In the end, 508 patients involved into the eventual data analyzing (eFigure). The smoking group had more male patients and a higher proportion of patients with alcohol consumption. Laboratory testing revealed the smoking group had higher levels of LDH, CRP, and WBC count, as well as their median and interquartile range. The two groups had no valid statistical discrepancy in terms of age, hypertension, hyperlipidemia, or cardiovascular disease history. The baseline characteristics of the smoking and non-smoking groups at admission are displayed in Table 1.

**Table 1:** Baseline characteristics of the study population by smoking history.

**Characteristics**	**Total patients (*n* = 508)**	**Smoking status**		***P*-value**
		**Never (*n* = 355)**	**Current (*n* = 153)**	
**Demographic**				
Age, years [median (Q1, Q3)]	52 (38, 6)	55 (38, 6)	48 (37, 6)	0.057
Male gender (*n*, %)	279 (55)	139 (39)	140 (92)	<0.001
Drinking history, (*n*, %)	90 (18)	25 (7)	65 (42)	<0.001
Hypertension, (*n*, %)	148 (29)	106 (30)	42 (27)	0.659
Diabetes, (*n*, %)	101 (20)	68 (19)	33 (22)	0.614
Hyperlipemia, (*n*, %)	31 (6)	20 (6)	11 (7)	0.638
Cardiovascular disease, (*n*, %)	63 (12)	44 (12)	19 (12)	0.994
**Laboratory findings**				
WBC count, × 10^9^/L, [median (Q1, Q3)]	12.57 (9.5, 15.9)	11.2 (9.0, 14.0)	14.85 (12.4, 18.0)	<0.001
RBC count, × 10^12^/L, (mean ± SD)	4.59 ± 0.7	4.48 ± 0.68	4.84 ± 0.67	<0.001
HGB level, × g/L, (mean ± SD)	143.12 ± 24.05	138.57 ± 23.14	153.67 ± 22.87	<0.001
PLT count, × 10^9^/L [median (Q1, Q3)]	226.5 (180.0, 277.5)	227 (182.0, 272.5)	224 (176.0, 280.0)	0.779
K+, × mmol/L [median (Q1, Q3)]	3.9 (3.6, 4.2)	3.9 (3.6, 4.2)	4 (3.7, 4.2)	0.081
Na+, × mmol/L, [median (Q1, Q3)]	138 (135.0, 141.0)	138 (136.0, 141.0)	138 (135.0, 140.0)	0.023
Ca+, × mmol/L, [median (Q1, Q3)]	2.22 (2.1, 2.3)	2.23 (2.1, 2.3)	2.19 (2.0, 2.3)	0.015
Cr, × μmol/L, [median (Q1, Q3)]	59 (47.7, 75.0)	56 (45.0, 72.0)	70 (56.0, 83.0)	<0.001
BG, × mmol/L, [median (Q1, Q3)]	8 (6.4, 11.6)	7.8 (6.3, 10.7)	8.6 (6.5, 13.6)	0.009
ALT, × U/L, [median (Q1, Q3)]	41 (23.0, 136.7)	43 (23.5, 139.0)	38 (23.0, 112.0)	0.343
LDH, × U/L, [median (Q1, Q3)]	283 (208.7,463.0)	269 (203.0, 443.5)	305 (218.0, 529.0)	0.032
TBIL, × μmol/L, [median (Q1, Q3)]	19 (13.0, 35.0)	19 (12.0, 33.0)	20 (15.0, 35.0)	0.047
ALB, × g/L, [median (Q1, Q3)]	41 (37.0, 44.0)	41 (37.3, 44.0)	41.1 (36.0, 44.0)	0.539
AMY, × U/L, [median (Q1, Q3)]	609 (200.7, 1,359.5)	611 (197.5, 1,344.0)	604 (248.0, 1,491.0)	0.928
LPS, × U/L, [median (Q1, Q3)]	2,148 (1,005.7, 4,336.2)	2,081 (901.5, 4,262.0)	2,318 (1,282.0, 4,535.0)	0.239
D-Dimer, × μg/ml, [median (Q1, Q3)]	7.5 (2.8, 16.2)	7.4 (2.9, 16.2)	7.6 (2.5, 16.5)	0.522
CRP, × mg/ml, [median (Q1, Q3)]	52.7 (9.4, 127.5)	38.9 (8.2, 122.0)	66.7 (15.6, 142.0)	0.003
PT, × second, [median (Q1, Q3)]	13.2 (12.5, 14.4)	13.1 (12.4, 14.2)	13.5 (12.8, 15.1)	0.019
APTT, × second, [median (Q1, Q3)]	30.3 (26.9,34.1)	30 (26.7, 33.5)	31.5 (27.4, 35.1)	0.012

SD, standard deviation; WBC, white blood cell count; RBC, red blood cell count; HGB, hemoglobin level; PLT, platelet; K+, potassium; Na+, sodium; Ca+, calcium; Cr, creatinine; BG, blood glucose; ALT, glutamic-pyruvic transaminase; LDH, lactate dehydrogenase; TBIL, total bilirubin; ALB, albumin; AMY, amylase; LPS, lipase; CRP, C-reactive protein; PT, prothrombin Time; APTT, activated partial thromboplastin time.

### Multivariable analysis and propensity score matching

To explore the relationship between the 27 factors and “MSAP or SAP”, we conducted single-factor and multi-factor analyses (Table 2). After adjusting for any confounding variables, we found that seven factors emerged as independent predictors of “MSAP or SAP”: smoking, WBC, HGB level, BG, Ca^+^ AMY, and PT. Even after adjusting for other variables, these factors remained statistically significant (*P* < 0.05).

**Table 2:** Univariable and multivariable analysis for “MSAP or SAP” in AP patients.

**Variables**	**Univariable**			**Multivariable**		
	**OR**	**95%CI**	***P*** **-value**	**OR**	**95%CI**	***P*** **-value**
**Demographic**						
Age	1.00	0.99–1.01	0.998	<NA>	<NA>	<NA>
Male gender	1.44	0.97–2.14	0.065	<NA>	<NA>	<NA>
Smoking	5.86	3.85–8.94	<0.001	5.01	2.76–9.09	<0.001
Drinking history	1.58	0.97–2.55	0.061	<NA>	<NA>	<NA>
Hypertension,	1.13	0.74–1.72	0.551	<NA>	<NA>	<NA>
Diabetes	1.88	1.19–2.97	0.006	1.37	0.65–2.88	0.395
Hyperlipemia	1.13	0.74–1.72	0.551	<NA>	<NA>	<NA>
Cardiovascular disease	1.29	0.73–2.27	0.369	<NA>	<NA>	<NA>
**Laboratory findings**						
WBC count	1.25	1.19–1.32	<0.001	1.21	1.12–1.29	<0.001
RBC count	1.58	1.19–2.11	0.002	1.26	0.65–2.43	0.485
HGB level	1.02	1.00–1.02	0.005	0.97	0.96–0.99	0.016
PLT count	1.00	1.00–1.01	0.014	1.00	0.99–1.01	0.375
K+	1.64	1.16–2.32	0.005	1.24	0.68–2.29	0.474
Na+	0.99	0.97–1.01	0.811	<NA>	<NA>	<NA>
Ca+	0.01	0.01–0.38	<0.001	0.01	0.00–0.04	<0.001
Cr	1.00	1.00–1.01	0.001	1.00	0.99–1.01	0.380
BG	1.17	1.11–1.23	<0.001	1.12	1.04–1.21	0.001
ALT	1.00	0.99–1.00	0.660	<NA>	<NA>	<NA>
LDH	1.00	1.00–1.01	0.001	1.00	1.00–1.00	0.414
TBIL	1.01	1.00–1.01	0.030	1.00	0.99–1.01	0.415
ALB	0.91	0.88–0.95	<0.001	1.02	0.96–1.08	0.486
AMY	1.00	1.00–1.00	0.043	1.00	1.00–1.00	0.039
LPS	1.00	1.00–1.00	0.149	<NA>	<NA>	<NA>
D-Dimer	1.00	0.99–1.00	0.943	<NA>	<NA>	<NA>
CRP	1.01	1.00–1.00	<0.001	1.00	0.99–1.00	0.295
PT	1.21	1.10–1.34	<0.001	1.14	1.02–1.29	0.021
APTT	1.04	1.01–1.07	0.010	1.00	0.95–1.04	0.985

*P*-value is from univariable and multivariable (*P* < 0.05 indicates statistical significance).

Table 3 displays the baseline characteristics of the smoking and non-smoking groups of patients both before and after 1:1 PSM. A satisfactory balance between the smoking and non-smoking groups was attained by PSM, as most variables had standardized mean differences of <0.1.

**Table 3:** Patient baseline characteristics before and after PSM by smoking history.

**Variables**	**Before PSM**			**After PSM**		
	**Never smoking (*n* = 355)**	**Current smoking (*n* = 153)**	**SMD**	**Never smoking (*n* = 95)**	**Current smoking (*n* = 95)**	**SMD**
**Demographic**						
Age, × years [mean (SD)]	53.73 (18.03)	50.79 (17.93)	0.163	50.21 (18.35)	50.98 (17.90)	0.042
Male gender (*n*, %)	139 (39.2)	140 (91.5)	1.317	85 (89.5)	83 (87.4)	0.066
Drinking history (*n*, %)	25 (7.0)	65 (42.5)	0.900	24 (25.3)	27 (28.4)	0.071
Hypertension (*n*, %)	106 (29.9)	42 (27.5	0.053	24 (25.3)	23 (24.2	0.024
Diabetes (*n*, %)	68 (19.2)	33 (21.6)	0.060	16 (16.8)	15 (15.8)	0.028
Hyperlipemia (*n*, %)	20 (5.6)	11 (7.2)	0.06	3 (3.2)	2 (2.1	0.066
Cardiovascular disease (*n*, %)	44 (12.4)	19 (12.4)	0.001	9 (9.5)	11 (11.6)	0.069
**Laboratory findings**						
WBC count, × 10^9^/L [mean (SD)]	11.89 (4.40)	15.52 (5.29)	0.748	14.09 (4.69)	14.23 (4.22)	0.033
RBC count, × 10^12/^L [mean (SD)]	4.48 (0.68)	4.84 (0.67)	0.531	4.83 (0.67)	4.80 (0.70)	0.045
HGB level, × g/L [mean (SD)]	138.57 (23.14)	153.67 (22.87	0.657	152.12 (20.83)	151.26 (23.13)	0.039
PLT count, × 10^9^/L [mean (SD)]	229.36 (73.11)	228.45 (76.22)	0.012	228.16 (63.73)	224.53 (71.37)	0.054
K^+^, × mmol/L [mean (SD)]	3.92 (0.48)	4.04 (0.69)	0.208	4.00 (0.53)	3.91 (0.53)	0.170
Na^+^, × mmol/L [mean (SD)]	137.97 (8.20)	136.52 (11.58)	0.145	136.29 (14.27)	136.31 (14.24)	0.001
Ca^+^, × mmol/L [mean (SD)]	2.21 (0.24)	2.15 (0.24)	0.267	2.19 (0.23)	2.17 (0.21)	0.083
Cr, × μmol/L [mean (SD)]	64.68 (49.15)	91.04 (117.40)	0.293	71.72 (32.56)	70.51 (24.92)	0.042
BG, × mmol/L [mean (SD)]	8.97 (4.01)	10.37 (5.06)	0.307	9.33 (3.92)	9.53 (4.81)	0.046
ALT, × U/L [mean (SD)]	127.09 (188.77)	129.16 (229.55)	0.010	98.81 (134.79)	119.64 (187.10)	0.128
LDH, × U/L [mean (SD)]	404.68 (359.59)	488.22 (546.71)	0.181	401.82 (330.68)	395.21 (319.08)	0.020
TBIL, × μmol/L [mean (SD)]	29.13 (31.80)	34.64 (38.44)	0.156	30.04 (26.74)	32.43 (37.95)	0.073
ALB, × g/L [mean (SD)]	40.56 (5.53)	39.85 (6.18)	0.122	40.23 (6.52)	40.27 (6.20)	0.006
AMY, × U/L [mean (SD)]	996.43 (1,096.61)	1,093.03 (1,300.16)	0.080	838.94 (996.26)	912.34 (1,022.56)	0.073
LPS, × U/L [mean (SD)]	3,063.51 (2,960.17)	3,132.59 (2,561.67)	0.025	2,707.83 (2,575.56)	2,805.33 (2,353.06)	0.040
D-Dimer, × μg/ml [mean (SD)]	15.05 (20.76)	16.93 (22.15)	0.088	15.45 (20.12)	17.77 (23.01)	0.107
CRP, × mg/ml [mean (SD)]	68.74 (75.35)	100.93 (107.91)	0.346	75.22 (84.66)	82.35 (97.02)	0.078
PT, × second [mean (SD)]	13.67 (2.77)	14.03 (2.45)	0.135	14.04 (3.70)	13.73 (1.82)	0.10
APTT, × second [mean (SD)]	30.76 (7.37	32.02 (6.89)	0.176	32.52 (11.61)	32.10 (6.06)	0.045

SMD, standardized mean difference, used to evaluate the balance before and after PSM ≥ 0.5 indicates imbalance; PSM, propensity score matching; SD, standard deviation; WBC, white blood cell count; RBC, red blood cell count; HGB, hemoglobin level; PLT, platelet; K+, potassium; Na+, sodium; Ca+, calcium; Cr, creatinine; BG, blood glucose; ALT, glutamic-pyruvic transaminase; LDH, lactate dehydrogenase; TBIL, total bilirubin; ALB, albumin; AMY, amylase; LPS, Lipase; CRP, C-reactive protein; PT, prothrombin time; APTT, activated partial thromboplastin time.

### The correlation between smoking pack-years and “MSAP or SAP”

**Figure 1:**
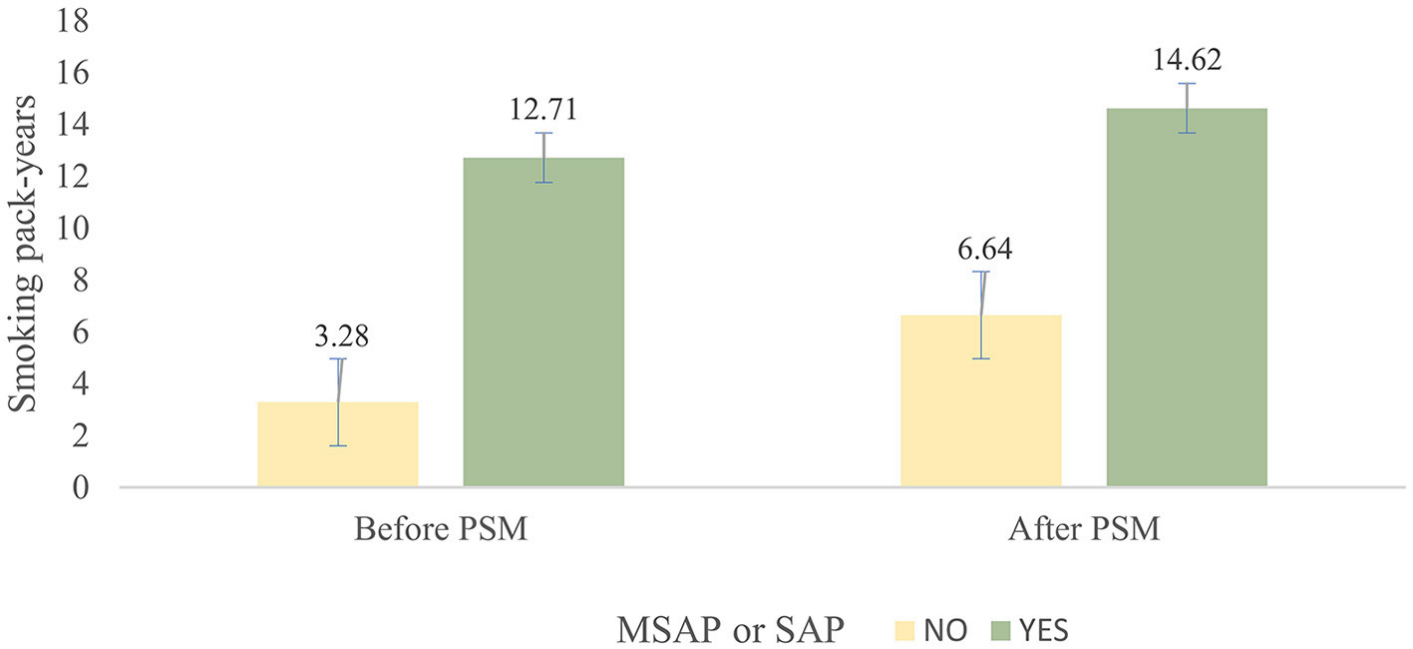
Mean and standard deviation of pack-years between the smoking group and non-smoking group before and after PSM.

The average smoking pack-years in the “MSAP or SAP” group were higher than those in the MAP group, both before PSM (3.28 vs. 12.71, *p* < 0.001) and after PSM (6.64 vs. 14.62, *p* < 0.001) (Figure 1). The proportion of patients with “MSAP or SAP” in the smoking group was larger than that in the non-smoking group, both before PSM (17.1 vs. 54.9%, *p* < 0.001) and after PSM (9.4 vs. 24.7%, *p* < 0.001) (Table 4). The risk of “MSAP or SAP” was higher in the smoking group than in the non-smoking group (unadjusted OR 5.86, 95% CI 3.85–8.94, *p* < 0.001). After adjusting for confounding factors, this result remained significant (adjusted OR 5.01, 95% CI 2.76–9.09, *p* < 0.001). With minimized selection bias by PSM, consistent finding was obtained (adjusted OR after PSM: 4.18, 95% CI 2.18–8.03, *p* < 0.001) (Table 5). Additionally, results from examining smoking pack-years as an ongoing factor were in coincidence: the unadjusted odds increased by 8% and the adjusted odds increased by 7% for every unit increase in smoking pack-years.

**Table 4:** Comparison of the incidence of “MSAP or SAP” after PSM based on smoking status.

**MSAP or SAP**	**Before PSM**		P^*^-value	**After PSM**		***P*-value**
	**Never smoking (*n* = 355)**	**Current smoking (*n* = 153)**		**Never smoking (*n* = 95)**	**Current smoking (*n* = 95)**	
Yes	61 (17.1%)	84 (54.9%)	<0.001	18 (9.4%)	47 (24.7%)	<0.001
No	294 (82.8%)	69 (45.0%)		77 (40.5%)	48 (25.2%)	

^*^P-value is from chi-squared test to indicate significant differentiation (*P* < 0.05 means significant differentiation).

**Table 5:** Comparison of the unadjusted and risk-adjusted OR by different smoking status.

**Type**	**Smoking status**	**Events *n* (%)**	**Unadjusted OR**	***P-*value**	**Multivariable regression adjusted OR**	***P-*value**	**PSM adjusted OR**	***P*-value**
Continuous	Per 1	NA	1.08 (1.05–1.10)	<0.001	1.07 (1.04–1.10)	<0.001	NA	NA
Cut off	Never	61 (3.3)	1 (Reference)	NA	1 (Reference)	NA	1 (Reference)	NA
	Current	84 (16.5)	5.86 (3.85–8.94)	<0.001	5.01 (2.76–9.09)	<0.001	4.18 (2.18–8.03)	<0.001
Smoking degree	Never	61 (3.3)	1 (Reference)	NA	1 (Reference)	NA	1 (Reference)	NA
	Light	15 (2.9)	2.89 (1.44–5.08)	0.003	3.76 (1.40–10.07)	0.008	1.72 (0.52–5.69)	0.374
	Moderate	34 (6.6)	6.30 (3.52–11.26)	<0.001	4.94 (2.23–10.92)	<0.001	2.63 (1.18–5.88)	0.018
	Heavy	35 (6.8)	9.37 (4.98–17.62)	<0.001	8.08 (3.39–19.25)	<0.001	3.25 (1.21–8.67)	0.019

The factors of the multivariable regression: age, gender, drinking history, hypertension, diabetes, hyperlipemia, cardiovascular disease, WBC count, RBC count, HGB level, PLT count, K+, Na+, Ca+, Cr, BG, ALT, LDH, TBIL, ALB, AMY, LPS, D-Dimer, CRP, PT, APTT.

CI, confidence interval; OR, odds ratio; PSM, propensity scores matching.

Compared to non-smoking patients, after adjusting for confounding factors, patients with varying degrees of smoking (light to heavy) exhibited an increased risk of developing “MSAP or SAP” (light: adjusted OR 3.76, 95% CI 1.40–10.07, *p* = 0.008; moderate: adjusted OR 4.94, 95% CI 2.23–10.92, *p* < 0.001; heavy: adjusted OR 8.08, 95% CI 3.39–19.25, *p* < 0.001). This linkage persisted even after PSM (light: adjusted OR after PSM 1.72, 95% CI 0.52–5.69, *p* = 0.374; moderate: adjusted OR after PSM 2.63, 95% CI 1.18–5.88, *p* = 0.018; heavy: adjusted OR after PSM 3.25, 95% CI 1.21–8.67, *p* = 0.019) (Table 5).

**Figure 2:**
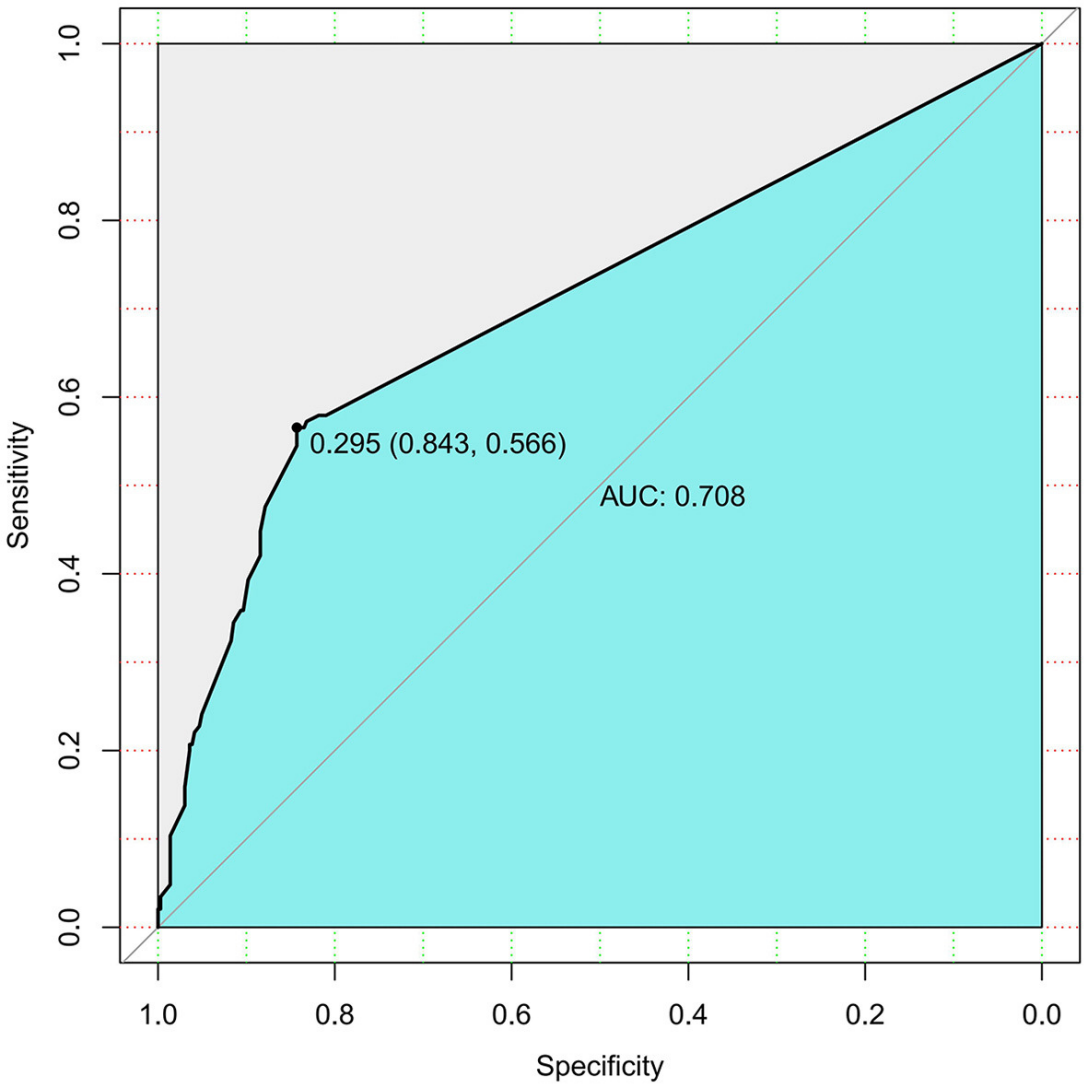
Smoking history prediction model for “MASP or SAP” rate in AP (AUC).

Additionally, the AUC of the ROC curve for smoking predicting the severity of AP was 0.708 (Figure 2), showing a risk of “MSAP or SAP” with a moderate predictive value.

### Dose-response linkage and interaction analysis

**Figure 3:**
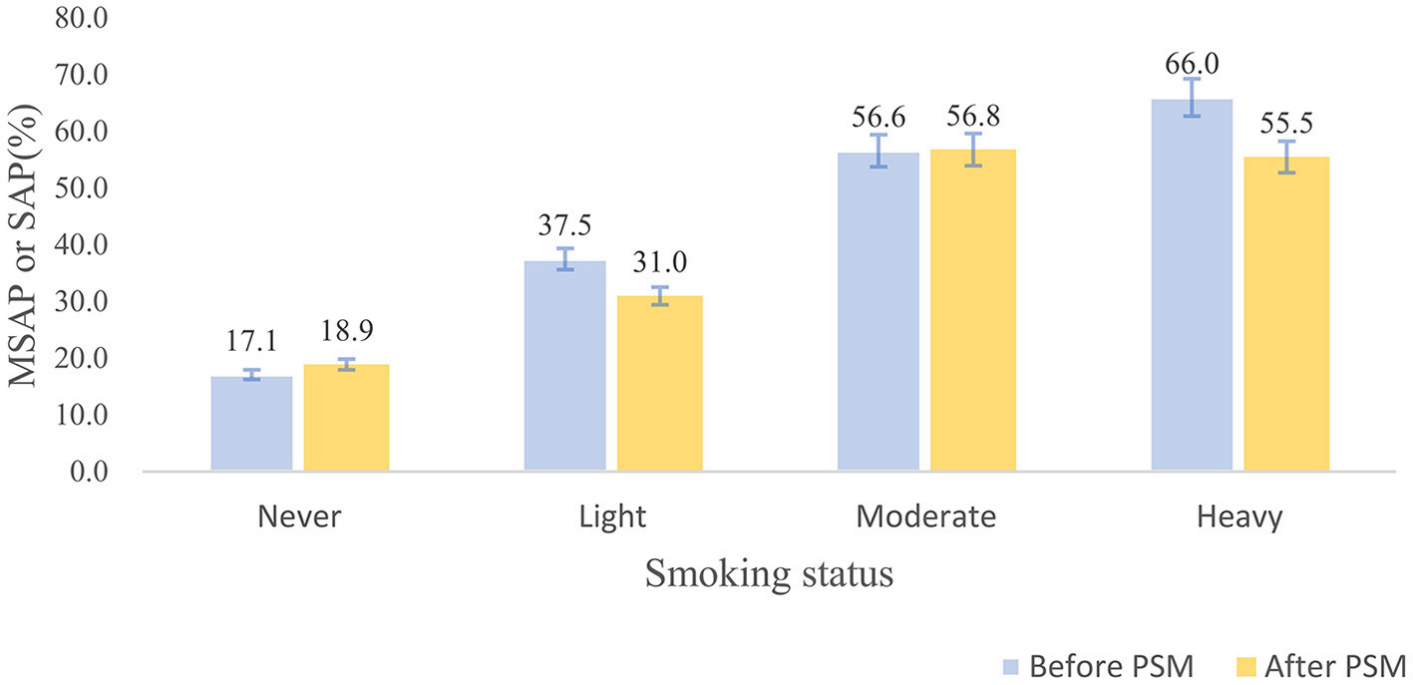
Rate of “MSAP or SAP” in each group by smoking status (never, light, moderate, and heavy smoking group).

**Figure 4:**
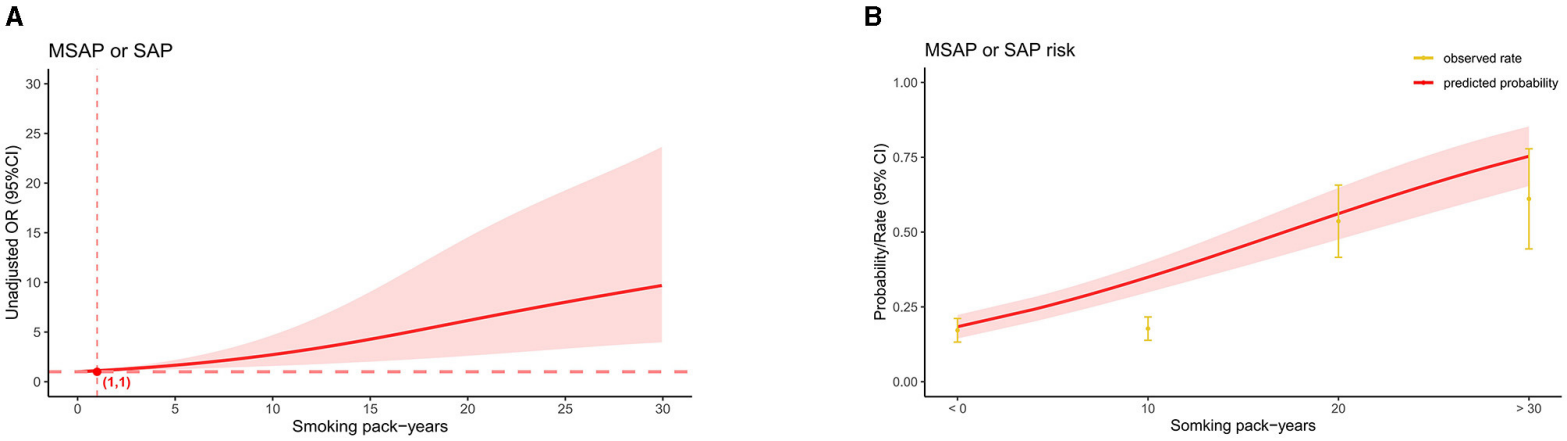
Linkage between smoking pack-years and “MASP or SAP” in patients with AP before PSM. **(A)** Adjusted odds ratios (ORs) and 95% confidence intervals (CIs) are shown for every five smoking pack-years interval. **(B)** Predicted probabilities and the observed rate of “MASP or SAP”.

**Figure 5:**
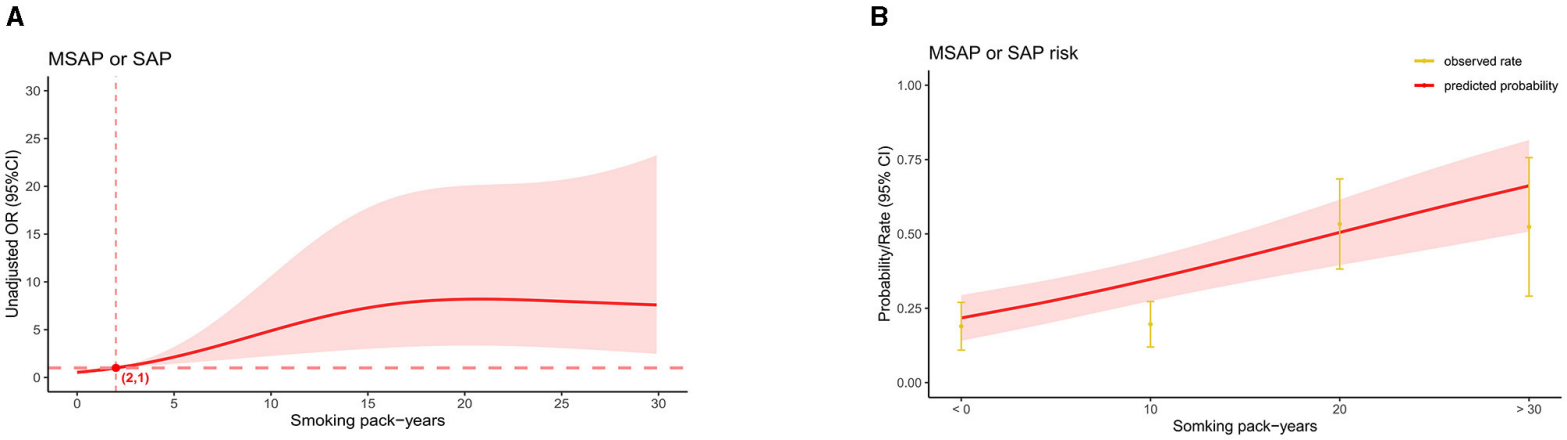
Linkage between smoking pack-years and “MASP or SAP” in patients with AP after PSM. **(A)** Adjusted odds ratios (ORs) and 95% confidence intervals (CIs) are shown for every five smoking pack-years interval. **(B)** Predicted probabilities and the observed rate of “MASP or SAP”.

**Figure 6:**
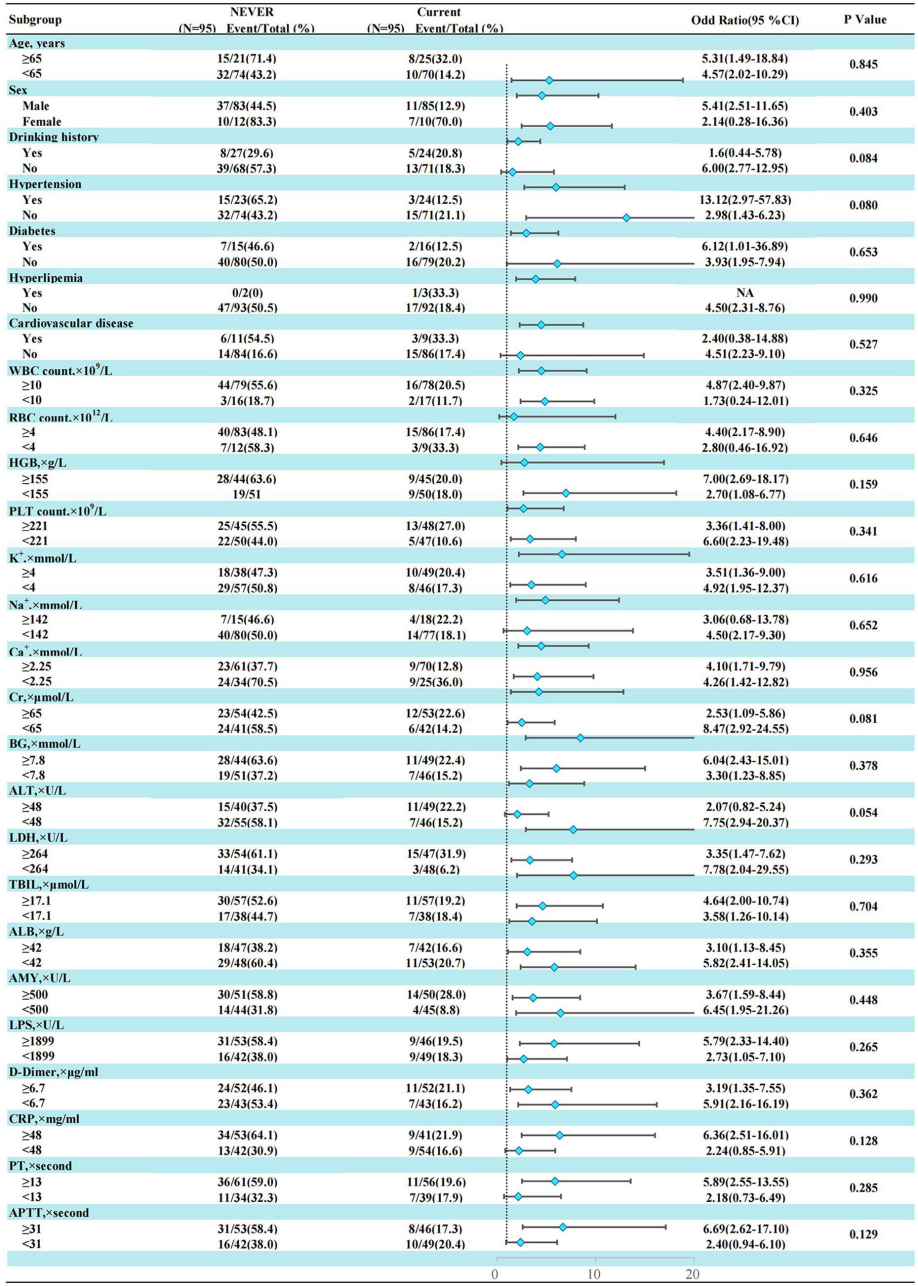
Subgroup analysis of smoking history and “MASP or SAP” in AP patients after propensity score matching.

In the AP patients studied, both before PSM (17.1% in non-smokers, 37.5% in light smokers, 56.6% in moderate smokers, and 66% in heavy smokers) and after PSM (18.9% in non-smokers, 31% in light smokers, 56.8% in moderate smokers, and 55.5% in heavy smokers), the incidence of “MSAP or SAP” increased with increasing smoking pack-years (Figure 3). Before and after PSM, there was a dose-response linkage between the risk of “MSAP or SAP” and smoking pack-years (before PSM: Figure 4; after PSM: Figure 5). Non-smokers had a lower risk of “MSAP or SAP” compared to smokers (Figures 4A, 5A). According to the level of smoking pack-years, the predicted and observed rates of MSAP or SAP increased with higher smoking pack-years, and the predicted and observed rates of MSAP or SAP were consistent (Figures 4B, 5B), indicating a dose-response relationship. Additionally, possible interactions between smoking and other factors were assessed in this study (Figure 6). There were no discernible interactions between smoking and the other factors (*p* > 0.05). Colinearity analysis shows that no collinearity exists between variables (eTable). All in all, these findings suggest that smoking is an independent risk factor for exacerbating AP.

## Discussion

In our study, we found that smoking is independently associated with the severity of AP. Compared to AP patients who have never smoked, smokers with AP have a more severe condition and a higher incidence of “MSAP or SAP”. Moreover, there is a dose-response linkage between smoking pack-years and the risk of “MSAP or SAP”, with the risk increasing with higher smoking pack-years. Even accounting for possible demographic and clinical variables, the association between smoking and the occurrence of “MSAP or SAP” remained significant. This suggests that smoking itself exacerbates AP. PSM also yielded consistent results, confirming that the linkage between smoking and the severity of pancreatitis cannot be attributable solely to baseline variation within the two groups.

Previous studies have indicated that smoking increases the risk of AP. A cohort study by Tolstrup et al. (10), involving 17,905 patients, observed that 46% of AP cases during the follow-up period could be attributed to smoking. They noted a dose-response relationship between smoking pack-years (15–29 pack-years) and the hazard ratio (HR) for AP (HR 2.2; 95% CI 1.2–3.8). Similarly, a large-scale study by Lee et al. (16) (*N* = 4,238,822) found that compared to non-smokers, current smokers had an adjusted HR of 1.66 (CI, 1.53–1.8) for acute pancreatitis, higher than that of former smokers (HR 1.34; CI, 1.17–1.54). China's largest prospective cohort study (17) (*n* = 512,891) also demonstrated an increased risk of AP among current male smokers (HR 1.45; 95% CI 1.28–1.64; *P* = 0.02). While aforesaid studies revealed that smoking increased the prevalence of AP, few have focused on the linkage between smoking and the severity of AP. Kim et al. (18) conducted a retrospective analysis of 905 AP patients and found that smoking was an independent risk factor for the development of SAP (OR: 7.22; 95% CI: 1.05–49.69; *P* = 0.04).

Several mechanisms may underlie the observed exacerbation of AP severity by smoking. Firstly, smoking directly damages pancreatic tissue through intricate mechanisms. Overall, chronic smoking exposure promotes pancreatic fibrosis, calcification, and chronic inflammation. This leads to premature activation of pancreatic enzymes and reduced secretion, causing retention of active digestive enzymes within acinar cells, exacerbating pancreatic autodigestion (14, 19, 20). This may increase the risk of complications in AP patients, such as pancreatic leakage, accumulation of necrotic pancreatic material, pseudocysts, and pancreatic abscesses. Additionally, pancreatic necrosis exacerbates systemic inflammation by releasing various inflammatory mediators into the bloodstream, potentially leading to multi-organ failure (21–24). For instance, Garg et al. (25) demonstrated a bidirectional relationship between the severity of pancreatic necrosis and organ failure. The extent of pancreatic necrosis influences the severity of organ failure, while organ failure exacerbates the progression of pancreatic necrosis. In a meta-analysis by Petrov et al. (26) based on 1,478 AP patients, it was found that the impact of organ failure and pancreatic necrosis on mortality was comparable; the presence of either indicates severe disease. Multi-organ dysfunction syndrome, extent of pancreatic necrosis, infection, and sepsis are major determinants of mortality in AP.

Secondly, ingredients of tobacco that can lead to inflammation in a number of different disorders. When acute pancreatitis (AP) occurs, this effect may exacerbate the severity of AP. For instance, a prospective study by Colak et al. (27) involving 98,085 participants found that plasma C-reactive protein levels increased by 4.8% (95% CI 4.4–5.2%) for every 10 pack-years and by 1.6% (95% CI 0.4–2.8%) for each T allele, indicating that both genetically and observably, greater tobacco use is linked to increased systemic inflammation. According to Liu et al. (28), nicotine can cause inflammatory reactions by triggering signaling pathways including STAT3 and NF-κB that are linked to inflammation. This causes increased intracellular inflammation, inflammatory cells to be recruited, inflammatory mediators to be induced, and tissue damage and inflammation to worsen. Nicotine also can activate AChR expressed on immune cell surfaces, thereby reducing the immunological response and blocking macrophages and lymphocytes from functioning (29). This may make inflammation and infection more likely.

Lastly, Smoking itself can act as a risk factor for various organ diseases (30), such as chronic obstructive pulmonary disease (COPD), atherosclerosis, coronary artery disease, myocardial infarction, heart failure, and stroke. Moreover, both moderately MSAP and SAP can result in varying degrees of organ failure. Prolonged exposure to tobacco may exacerbate this process, leading to an increased risk of susceptible organ infections and failure. For instance, NNK in tobacco suppresses the production of IL-8, which plays a crucial role in acute inflammation by recruiting and activating neutrophils. A reduction in IL-8 may lead to an increased incidence of pulmonary infections (31, 32). Munzel et al. (33) reported that smoking can lead to endothelial dysfunction, increased oxidative stress, and cardiovascular events.

It's worth noting that in our study, the increase in risk and incidence rate of “MSAP or SAP” after PSM exhibits a lower inflection point compared to pre-PSM (1 pack-year before PSM, 2 pack-years after PSM). However, irrespective of pre or post PSM, when smoking pack-years are ≤ 2, the corresponding OR values remain close to 1, consistent with our post-PSM findings regarding the risk of “MSAP or SAP” (smoking pack-years ≤ 10, OR 1.72, 95% CI 0.52–5.69, *P* = 0.374). This suggests that at lower levels of smoking pack-years, the risk of “MSAP or SAP” occurrence isn't significantly different from that of non-smokers. Smoking needs a certain accumulation time to noticeably exacerbate the severity of AP, with the specific threshold currently unknown. Similar cumulative effects of smoking have been documented in other studies. For instance, a comprehensive study by Tolstrup et al. (10) revealed no significant difference in pancreatitis incidence between smokers consuming 1–14 g/day and never-smokers, yielding a HR of 1.5 (95% CI 0.9–2.5). Pancreatitis incidence attained statistical significance only with increased smoking to 15–24 g/day, showing an HR of 2.5 (95% CI 1.5–3.9). Similarly, Hansen et al. (34), based on a prospective study of 108,438 individuals, reported HRs of 1.1 (95% CI 0.8–1.7, with no statistical significance) and 3.6 (95% CI 1.8–2.5) for smoking pack-years of 0.1–9 and 9.1–24, respectively, for chronic pancreatitis risk. Large-scale prospective studies are imperative to elucidate the underlying mechanisms and determine the threshold for cumulative effects.

Alcohol is considered an independent risk factor for the occurrence of AP (35). According to calculations based on weekly alcohol consumption, drinking ≥5 drinks per day significantly increases the risk of developing AP (36, 37). However, in our study, we did not find a significant correlation between a history of alcohol consumption and the severity of AP (*P* = 0.061). On one hand, alcohol consumption may be a factor in the onset of pancreatitis rather than exacerbating its severity. On the other hand, this lack of correlation may be related to the drinking patterns of the patients included in our study. We observed a higher proportion of occasional drinkers rather than daily alcohol abusers among our patients. Additionally, in our study, we did not find a significant interaction between a history of alcohol consumption and smoking (*P* = 0.084).

This study boasts several strengths: we employed propensity score matching and multivariable logistic regression to rigorously control for confounding factors. The dose-response linkage analysis visually depicted the correlation between smoking pack-years and the severity of AP. Nonetheless, certain limitations should be acknowledged: the sample size was relatively small; the single-center retrospective design precludes establishing causality, necessitating further prospective and multicenter studies to validate these findings; our study only included non-smokers and current smokers, excluding former smokers, which limits our ability to determine whether smoking cessation can mitigate the exacerbating effect of smoking on the severity of AP. Additionally, Relative confounding from unmeasured factors may continue even after corrections.

## Conclusion

In conclusion, our study provides evidence suggesting that smoking is associated with an increased risk of developing “MSAP or SAP” in patients with AP. We also observed a dose-response relationship between smoking pack-years and the severity of pancreatic involvement, indicating that the impact of smoking on the severity of AP may require a certain amount of time to accumulate before becoming evident. Our findings lay the groundwork for future longitudinal studies. However, whether smoking cessation can mitigate this effect requires further confirmation through new multicenter prospective randomized studies.

## Data availability statement

The original contributions presented in the study are included in the article/Supplementary material, further inquiries can be directed to the corresponding author.

## Ethics statement

The studies involving humans were approved by the Ethics Committee of Dandong Central Hospital. The studies were conducted in accordance with the local legislation and institutional requirements. Written informed consent for participation was not required from the participants or the participants' legal guardians/next of kin in accordance with the national legislation and institutional requirements.

## Author contributions

RL: Supervision, Writing – original draft, Writing – review & editing. WT: Data curation, Writing – original draft, Writing – review & editing. SY: Conceptualization, Supervision, Writing – original draft. XY: Methodology, Writing – original draft. LH: Project administration, Writing – original draft.
