# Supplementary material for: A dose–response correlation between smoking and severity of acute pancreatitis: a propensity score-matched study
Source: Front Med (Lausanne). 2024 Jul 29;11:1397111. doi: 10.3389/fmed.2024.1397111 (PMC11317375; doi:10.3389/fmed.2024.1397111)
Supplement: Supplementary file 1 [file Table_1.DOCX]

| **Variables** | **Before PSM** | | | **After PSM** | | |
| --- | --- | --- | --- | --- | --- | --- |
|  | **Never Smoking**  **(n=355)** | **Current Smoking**  **(n=40)** | **SMD** | **Never Smoking**  **(n=29)** | **Current Smoking**  **(n=29)** | **SMD** |
| **Demographic** |  |  |  |  |  |  |
| Age, ×years [mean (SD)] | 53.73 (18.03) | 45.05 (18.54) | 1.610 | 50.14 (15.75) | 45.79 (17.76) | 0.259 |
| Male gender (n, %) | 139 (39.2) | 39 (97.5) | 1.317 | 27 (93.1) | 28 (96.6) | 0.156 |
| Drinking history (n, %) | 25 (7.0) | 20 (50.0) | 1.082 | 11 (37.9) | 11 (37.9) | <0.001 |
| Hypertension (n, %) | 106 (29.9) | 11 (27.5) | 0.052 | 7 (24.1) | 8 (27.6) | 0.079 |
| Diabetes (n, %) | 68 (19.2) | 11 (27.5) | 0.198 | 8 (27.6) | 5 (17.2) | 0.250 |
| Hyperlipemia (n, %) | 20 (5.6) | 3 (7.5) | 0.075 | 1 (3.4) | 1 (3.4) | <0.001 |
| Cardiovascular disease (n, %) | 44 (12.4) | 3 (7.5) | 0.164 | 2 (6.9) | 2 (6.9) | <0.001 |
| **Laboratory findings** |  |  |  |  |  |  |
| WBC count, ×10^9^/L [mean (SD)] | 11.89(4.40) | 15.42 (5.25) | 0.730 | 13.65 (4.21) | 13.70 (3.59) | 0.011 |
| RBC count, ×10^12/^L [mean (SD)] | 4.48 (0.68) | 5.06 (0.63) | 0.880 | 5.01 (0.48) | 5.04 (0.62) | 0.061 |
| HGB level, ×g/L [mean (SD)] | 138.57 (23.14) | 157.82 (21.36) | 0.865 | 155.28 (14.17) | 157.17 (22.32) | 0.101 |
| PLT count, ×10^9^/L [mean (SD)] | 229.36(73.11) | 245.15 (57.96) | 0.239 | 232.21 (50.02) | 237.93 (53.53) | 0.110 |
| K^+^, ×mmol/L [mean (SD)] | 3.92(0.48) | 3.99 (0.55) | 0.141 | 3.97 (0.65) | 3.92 (0.45) | 0.104 |
| Na^+^, ×mmol/L [mean (SD)] | 137.97(8.20) | 137.32 (3.57) | 0.101 | 138.41 (3.78) | 137.66 (3.38) | 0.212 |
| Ca^+^, ×mmol/L [mean (SD)] | 2.21(0.24) | 2.25 (0.17) | 0.174 | 2.20 (0.28) | 2.23 (0.18) | 0.147 |
| Cr, ×μmol/L [mean (SD)] | 64.68(49.15) | 96.03 (151.74) | 0.278 | 70.83 (22.76) | 71.93 (22.38) | 0.049 |
| BG, ×mmol/L [mean (SD)] | 8.97(4.01) | 9.98 (4.44) | 0.238 | 8.56 (3.02) | 9.23 (4.21) | 0.184 |
| ALT, ×U/L [mean (SD)] | 127.09(188.77) | 133.82 (241.19) | 0.031 | 154.55 (284.21) | 123.90 (222.15) | 0.120 |
| LDH, ×U/L [mean (SD)] | 404.68(359.59) | 417.42 (541.53) | 0.028 | 354.10 (286.40) | 449.45 (621.45) | 0.197 |
| TBIL, ×μmol/L [mean (SD)] | 29.13(31.80) | 29.52 (32.59) | 0.012 | 32.24 (26.88) | 29.00 (34.22) | 0.105 |
| ALB, ×g/L [mean (SD)] | 40.56(5.53) | 41.91 (4.46) | 0.269 | 40.83 (5.84) | 41.84 (4.35) | 0.196 |
| AMY, ×U/L [mean (SD)] | 996.43(1096.61) | 841.95 (984.49) | 0.148 | 982.52 (1056.50) | 744.97 (913.95) | 0.240 |
| LPS, ×U/L [mean (SD)] | 3063.51(2960.17) | 2820.50 (2188.20) | 0.093 | 2976.72(2378.14) | 2722.66 (2107.20) | 0.113 |
| D-Dimer, ×μg/ml [mean (SD)] | 15.05(20.76) | 14.68 (17.90) | 0.019 | 14.31 (18.06) | 16.33 (19.62) | 0.107 |
| CRP, ×mg/ml [mean (SD)] | 68.74(75.35) | 79.20 (95.30) | 0.122 | 59.10 (64.49) | 73.61 (91.68) | 0.183 |
| PT, ×second [mean (SD)] | 13.67(2.77) | 13.55 (1.68) | 0.051 | 14.17 (1.82) | 13.70 (1.56) | 0.279 |
| APTT, ×second [mean (SD)] | 30.76(7.37 | 31.84 (5.84) | 0.161 | 33.43 (17.43) | 30.99 (3.78) | 0.194 |

**eTables**

eTable 1. The baseline characteristics of non-smokers and patients with ≤10 pack-years of smoking history before and after PSM.

| **Variables** | **Before PSM** | | | **After PSM** | | |
| --- | --- | --- | --- | --- | --- | --- |
|  | **Never Smoking**  **(n=355)** | **Current Smoking**  **(n=60)** | **SMD** | **Never Smoking**  **(n=51)** | **Current Smoking**  **(n=51)** | **SMD** |
| **Demographic** |  |  |  |  |  |  |
| Age, ×years [mean (SD)] | 53.73 (18.03) | 49.48 (15.04) | 0.256 | 49.72 (16.16) | 49.95 (16.58) | 0.014 |
| Male gender (n, %) | 139 (39.2) | 49 (81.7) | 0.965 | 33 (84.6) | 30 (76.9) | 0.196 |
| Drinking history (n, %) | 25 (7.0) | 19 (31.7) | 0.656 | 10 (25.6) | 11 (28.2) | 0.058 |
| Hypertension (n, %) | 106 (29.9) | 14 (23.3) | 0.148 | 7 (17.9) | 8 (20.5) | 0.065 |
| Diabetes (n, %) | 68 (19.2) | 13 (21.7) | 0.062 | 7 (17.9) | 4 (10.3) | 0.222 |
| Hyperlipemia (n, %) | 20 (5.6) | 4 (6.7) | 0.043 | 4 (10.3) | 3 (7.7) | 0.090 |
| Cardiovascular disease (n, %) | 44 (12.4) | 8 (13.3) | 0.028 | 5 (12.8) | 4 (10.3) | 0.080 |
| **Laboratory findings** |  |  |  |  |  |  |
| WBC count, ×10^9^/L [mean (SD)] | 11.89(4.40) | 15.26 (5.10) | 0.709 | 14.98 (4.26) | 15.42 (4.91) | 0.094 |
| RBC count, ×10^12/^L [mean (SD)] | 4.48 (0.68) | 4.70 (0.71) | 0.306 | 4.75 (0.61) | 54.85 (0.63) | 0.170 |
| HGB level, ×g/L [mean (SD)] | 138.57 (23.14) | 151.53 (25.32) | 0.534 | 152.72 (22.51) | 155.74 (22.14) | 0.136 |
| PLT count, ×10^9^/L [mean (SD)] | 229.36(73.11) | 233.07 (75.44) | 0.050 | 234.21 (69.74) | 228.74 (65.21) | 0.081 |
| K^+^, ×mmol/L [mean (SD)] | 3.92(0.48) | 4.06 (0.83) | 0.199 | 3.96 (0.49) | 3.93 (0.60) | 0.051 |
| Na^+^, ×mmol/L [mean (SD)] | 137.97(8.20) | 135.03 (17.81) | 0.212 | 133.69 (21.65) | 134.03 (21.83) | 0.015 |
| Ca^+^, ×mmol/L [mean (SD)] | 2.21(0.24) | 2.11 (0.28) | 0.401 | 2.12 (0.30) | 2.15 (0.24) | 0.092 |
| Cr, ×μmol/L [mean (SD)] | 64.68(49.15) | 73.78 (39.76) | 0.204 | 65.31 (18.46) | 69.05 (24.15) | 0.174 |
| BG, ×mmol/L [mean (SD)] | 8.97(4.01) | 10.52 (4.75) | 0.352 | 9.93 (4.07) | 9.83 (4.45) | 0.025 |
| ALT, ×U/L [mean (SD)] | 127.09(188.77) | 107.20 (171.30) | 0.110 | 106.44 (171.61) | 107.62 (181.57) | 0.007 |
| LDH, ×U/L [mean (SD)] | 404.68(359.59) | 468.43 (488.54) | 0.149 | 416.95 (293.13) | 308.64 (169.73) | 0.452 |
| TBIL, ×μmol/L [mean (SD)] | 29.13(31.80) | 26.58 (20.09) | 0.096 | 25.12 (17.77) | 24.08 (19.07) | 0.057 |
| ALB, ×g/L [mean (SD)] | 40.56(5.53) | 39.50 (6.89) | 0.170 | 39.07 (5.26) | 40.29 (6.60) | 0.205 |
| AMY, ×U/L [mean (SD)] | 996.43(1096.61) | 1217.67 (1477.07) | 0.170 | 881.67 (906.45) | 976.08 (1112.25) | 0.093 |
| LPS, ×U/L [mean (SD)] | 3063.51(2960.17) | 3177.28 (2616.82) | 0.041 | 2907.38 (2389.95) | 2758.23(2194.61) | 0.065 |
| D-Dimer, ×μg/ml [mean (SD)] | 15.05(20.76) | 15.89 (22.63) | 0.038 | 17.95 (23.30) | 15.04 (22.33) | 0.128 |
| CRP, ×mg/ml [mean (SD)] | 68.74(75.35) | 103.64 (120.66) | 0.347 | 82.97 (95.94) | 95.14 (108.70) | 0.119 |
| PT, ×second [mean (SD)] | 13.67(2.77) | 14.01 (3.01) | 0.115 | 13.66 (1.79) | 14.38 (3.56) | 0.253 |
| APTT, ×second [mean (SD)] | 30.76(7.37 | 31.87 (8.00) | 0.144 | 31.47 (5.66) | 31.47 (5.66) | 0.016 |

eTable 2. The baseline characteristics of non-smokers and patients with >10 but ≤20 pack-years of smoking history before and after PSM.

eTable 3. The baseline characteristics of non-smoking patients were compared with those of patients with a smoking history of more than 20 pack-years before and after PSM.

| **Variables** | **Before PSM** | | | **After PSM** | | |
| --- | --- | --- | --- | --- | --- | --- |
|  | **Never Smoking**  **(n=355)** | **Current Smoking**  **(n=53)** | **SMD** | **Never Smoking**  **(n=36)** | **Current Smoking**  **(n=36)** | **SMD** |
| **Demographic** |  |  |  |  |  |  |
| Age, ×years [mean (SD)] | 53.73 (18.03) | 56.60 (19.07) | 0.155 | 55.11 (17.78) | 57.53 (18.75) | 0.132 |
| Male gender (n, %) | 139 (39.2) | 52 (98.1) | 1.646 | 34 (94.4) | 35 (97.2) | 0.139 |
| Drinking history (n, %) | 25 (7.0) | 26 (49.1) | 1.058 | 13 (36.1) | 14 (38.9) | 0.057 |
| Hypertension (n, %) | 106 (29.9) | 17 (32.1) | 0.048 | 9 (25.0) | 10 (27.8) | 0.063 |
| Diabetes (n, %) | 68 (19.2) | 9 (17.0) | 0.057 | 5 (13.9) | 5 (13.9) | <0.001 |
| Hyperlipemia (n, %) | 20 (5.6) | 4 (7.5) | 0.077 | 1 (2.8) | 2 (5.6) | 0.139 |
| Cardiovascular disease (n, %) | 44 (12.4) | 8 (15.1) | 0.078 | 4 (11.1) | 5 (13.9) | 0.084 |
| **Laboratory findings** |  |  |  |  |  |  |
| WBC count, ×10^9^/L [mean (SD)] | 11.89(4.40) | 15.90 (5.60) | 0.797 | 14.48(3.94) | 13.61 (3.48) | 0.234 |
| RBC count, ×10^12/^L [mean (SD)] | 4.48 (0.68) | 4.84 (0.62) | 0.550 | 4.88 (0.70) | 4.75 (0.58) | 0.203 |
| HGB level, ×g/L [mean (SD)] | 138.57 (23.14) | 152.96 (20.96) | 0.652 | 1148.36 (30.07) | 149.11 (18.12) | 0.030 |
| PLT count, ×10^9^/L [mean (SD)] | 229.36(73.11) | 210.62 (86.27) | 0.234 | 226.47 (68.88) | 205.31 (80.46) | 0.283 |
| K^+^, ×mmol/L [mean (SD)] | 3.92(0.48) | 4.07 (0.62) | 0.270 | 4.01 (0.64) | 4.05 (0.53) | 0.075 |
| Na^+^, ×mmol/L [mean (SD)] | 137.97(8.20) | 137.58 (4.30) | 0.058 | 138.42 (4.84) | 137.47 (3.97) | 0.214 |
| Ca^+^, ×mmol/L [mean (SD)] | 2.21(0.24) | 2.12 (0.23) | 0.406 | 2.19 (0.26) | 2.16 (0.21) | 0.110 |
| Cr, ×μmol/L [mean (SD)] | 64.68(49.15) | 106.81 (143.57) | 0.393 | 75.58 (22.00) | 80.78 (38.29) | 0.166 |
| BG, ×mmol/L [mean (SD)] | 8.97(4.01) | 10.51 (5.85 | 0.305 | 10.81 (5.46) | 9.79 (5.16) | 0.193 |
| ALT, ×U/L [mean (SD)] | 127.09(188.77) | 150.51 (275.81) | 0.099 | 120.31 (171.65) | 113.92 (138.73) | 0.041 |
| LDH, ×U/L [mean (SD)] | 404.68(359.59) | 564.06 (610.56) | 0.318 | 483.47 (424.71) | 433.75 (358.25) | 0.127 |
| TBIL, ×μmol/L [mean (SD)] | 29.13(31.80) | 47.61 (52.89) | 0.423 | 36.86 (32.49) | 36.29 (32.55) | 0.017 |
| ALB, ×g/L [mean (SD)] | 40.56(5.53) | 38.69 (6.16) | 0.320 | 39.94 (5.43) | 40.51 (4.93) | 0.110 |
| AMY, ×U/L [mean (SD)] | 996.43(1096.61) | 1141.43 (1292.32) | 0.121 | 1159.44(1455.99) | 1207.92(1249.40) | 0.036 |
| LPS, ×U/L [mean (SD)] | 3063.51(2960.17) | 3317.55 (2776.50) | 0.089 | 3666.81 (3039.75) | 3326.50 (2138.63) | 0.129 |
| D-Dimer, ×μg/ml [mean (SD)] | 15.05(20.76) | 19.81 (24.47) | 0.210 | 19.93 (24.52) | 21.44 (27.11) | 0.059 |
| CRP, ×mg/ml [mean (SD)] | 68.74(75.35) | 114.24 (100.77) | 0.511 | 84.59 (84.82) | 84.45 (87.93) | 0.002 |
| PT, ×second [mean (SD)] | 13.67(2.77) | 14.40 (2.21) | 0.292 | 13.73 (2.13) | 13.78 (2.05) | 0.024 |
| APTT, ×second [mean (SD)] | 30.76(7.37 | 32.32 (6.34) | 0.227 | 33.71 (15.91) | 32.13 (6.54) | 0.130 |

| **Variables** | Hypertension | Diabetes | Hyperlipemia | Cardiovascular disease | CRP | PT | APTT |
| --- | --- | --- | --- | --- | --- | --- | --- |
| **Tolerance** | 0.907 | 0.964 | 0.970 | 0.914 | 0.976 | 0.844 | 0.840 |
| **VIF** | 1.103 | 1.037 | 1.031 | 1.094 | 1.024 | 1.185 | 1.190 |

eTable 4. Collinearity analysis by smoking.

Abbreviations: CRP (C-Reactive Protein); PT (Prothrombin Time); APTT (Activated Partial Thromboplastin Time).

**eFigure**: Flow chart of enrollment

929 patients with acute pancreatitis

in Dandong Central Hospital

421 patients exclude:

With other pancreatic diseases, such as chronic pancreatitis. (n=123)

With past admission due to AP. (n=92)

Incomplete or unavailable data. (n=86)

With other serious diseases, such as tumors, autoimmune diseases, etc. (n=52)

Taking medications that may affect the severity of pancreatitis, such as tetracycline. (n=47)

With history of pancreatic surgery. (n=21)

The study population eligible for inclusion.

(n=508)

8

Multivariate logistic regression:

Independent risk factor

The propensity score matching method

(n=190)

18 out of 95 patients with non-smoking history diagnosed with MAP

47 out of 95 patients with smoking history diagnosed with MSAP or SAP

Patients without Smoking history

(n=355)

Patients with MAP(n=294)

Patients with MSAP or SAP(n=61)

Patients with Smoking history

(n=153)

Patients with MAP(n=69)

Patients with MSAP or SAP(n=84)
